# From Day to Day to Year to Year: Developmental Antecedents and Outcomes of Changes in Emotion

**DOI:** 10.1093/geroni/igab046.2198

**Published:** 2021-12-17

**Authors:** Jeremy Hamm, Meaghan Barlow

## Abstract

Research shows that emotions play an important role in successful aging. However, less is known about how day-to-day fluctuations and multi-year changes in positive and negative emotions are implicated in adaptive development. Thus, the present studies address the developmental antecedents and outcomes of micro- and macro-longitudinal changes in different positive and negative emotions. Blöchl, Oertzen, and Kunzmann use 12-year data from the Health and Retirement Study to examine whether socioeconomic resources influence trajectories of positive emotion and physical functioning and their interrelations. Hamm, Wrosch, Barlow, and Kunzmann investigate psychosocial and health-related resources that predict two-year stability and change in adaptive and maladaptive daily patterns of calmness, excitement, sadness, and anger. Pauly et al. examine the extent to which health status moderates the association between daily fluctuations in seven affective states and corresponding changes in stress-related cortisol secretion. Turner, Mogle, Hill, Bhargava, and Rabin study how positive and negative emotions experienced in response to daily challenges (memory lapses) mediate the association between age-related challenges and life satisfaction in a coordinated analysis of two datasets. Finally, Barlow addresses the extent to which variations in daily experiences of positive and negative emotions exhibit age-differential associations with daily satisfaction with life (i.e., emotion globalizing). This symposium thus integrates new research on emotional aging and contributes to a deeper understanding of how adaptive development shapes and is shaped by day-to-day fluctuations and long-term changes in different emotions.

